# Introducing the TOP framework: a novel phenotyping solution for collaborative phenotype algorithm development and application

**DOI:** 10.1186/s13326-026-00364-7

**Published:** 2026-07-27

**Authors:** Christoph Beger, Dorothea Strobach, Ralph Schäfermeier, Franz Matthies, Konrad Höffner, Alexandr Uciteli

**Affiliations:** 1 Institute for Medical Informatics, Statistics and Epidemiology, Leipzig University, Härtelstraße 16-18, 04107 Leipzig, Germany; 2 Hospital Pharmacy and Doctoral Program Clinical Pharmacy, University Hospital Ludwig-Maximilians-University Munich, Munich, Germany

**Keywords:** Algorithms, Computable phenotype, Data-driven phenotyping, Electronic health records, Health information interoperability, Patient stratification

## Abstract

**Background:**

Phenotyping, the comprehensive assessment of observable characteristics, is essential for advancing medical understanding and personalised healthcare. However, traditional phenotyping methods are often manual, time-intensive, and limited in scope. To address these challenges, this work introduces the TOP Framework, a software suite that leverages a structured approach. It provides tools for the formal definition of phenotypes, their organisation into ontological classes, the creation of phenotype models for disease-specific knowledge representation, and the generation of phenotype queries for automated data retrieval and analysis. A dynamic phenotype algorithm integrates these modules to efficiently identify individuals meeting complex phenotypic criteria. The Model for End-Stage Liver Disease (MELD) score serves as a running example to illustrate the framework’s capabilities. Furthermore, this paper presents a preliminary evaluation of the TOP Framework’s user experience by means of the User Experience Questionnaire (UEQ), assessing its usability and suitability for researchers and clinicians.

**Results:**

The TOP Framework includes a robust implementation of a reasoner model for deriving complex phenotypes and an automated testing module to ensure reliability. The user experience evaluation yielded generally positive results on a scale from −3 to 3 (*n* = 11), with mean scores and 95% confidence intervals as follows: attractiveness, 1.50 (CI = (1.07, 1.93)); pragmatic quality, 1.38 (CI = (1.00, 1.77)); and hedonic quality, 1.40 (CI = (0.76, 2.03)).

**Conclusions:**

The TOP Framework offers a novel and automated approach to phenotyping, with the potential to enhance the efficiency, scalability, and reproducibility of phenotyping studies. This advancement contributes to a deeper understanding of disease and the progression of precision medicine.

## Background

Phenotyping is a fundamental component of modern medicine and biological research and involves the comprehensive assessment of an individual’s observable characteristics or traits – including physical attributes, physiological aspects, biochemical markers, and behavioural patterns [1, 2]. A thorough understanding of phenotypes is essential for deciphering the complexities of diseases, enabling precise patient stratification for targeted therapeutic interventions, and advancing the paradigm of precision medicine [3].

Traditionally, the process of phenotyping has often relied on meticulous manual data collection and expert interpretation of diverse information sources, such as clinical notes, laboratory results, imaging studies, and questionnaires. Manual phenotyping approaches, while important, are inherently time-consuming, resource-intensive, and prone to interobserver variability [4]. These traditional methods also face significant challenges in integrating and analysing large, diverse datasets. Recent work highlights that the increasing availability of vast biomedical data describing patient phenotypes and treatments holds far greater research potential than is currently realised [5, 6]. In particular, the mining of electronic health records (EHR) can facilitate novel patient stratification and uncover new disease correlations, underscoring the need for automated frameworks that can fully leverage biomedical data for research and precision medicine [5].

The process of phenotyping typically involves the development and application of computable phenotypes. These are precise, algorithmic definitions of clinical conditions or characteristics that can be automatically identified and extracted from electronic health records [7]. Mo et al. [7] have proposed *desiderata* (goals) that should be addressed when developing computable representations of EHR-based phenotype algorithms, emphasising the need for robust and standardised approaches [8]. Furthermore, the concept of phenotype libraries, which are organised collections of these computable definitions, has emerged as a crucial resource for facilitating reproducible research (e.g., PheKB [9], HDR UK Phenotype Library [10]). Chapman et al. [11] have contributed significantly to understanding the landscape of phenotype definitions in EHRs and the requirements, challenges, and opportunities associated with building and utilising comprehensive phenotype libraries. However, current approaches often struggle with data heterogeneity and semantic inconsistencies [12] as well as the need for close collaboration between domain and IT experts [13].

Building upon this foundational work in defining and organising computable phenotypes and addressing mentioned limitations, this paper introduces the TOP Framework, developed by the Junior Research Group “Terminology- and Ontology-based Phenotyping (TOP)” within the SMITH consortium [14] of the German Medical Informatics Initiative (MII). The TOP Framework leverages advancements in data integration, semantic modelling, and automated reasoning to facilitate efficient and comprehensive phenotyping. Its key distinction lies in the separation of concerns between domain experts (e.g., physicians, pharmacologists, and pharmacists), who develop specifications of computable phenotypes (phenotype models) [7, 11], and IT experts, who handle the technical implementation of phenotype algorithms. This separation, facilitated by the framework’s structured approach for defining, modelling, and querying phenotypes through interconnected modules, enables the automated extraction of rich phenotypic insights from heterogeneous data sources without knowledge about the underlying query language [13, 15]. These modules include the formal definition of phenotypes, the organisation of phenotypes into ontological classes, the creation of phenotype models to represent disease-specific knowledge, and the generation of executable queries for automated data retrieval and analysis. The Model for End-Stage Liver Disease (MELD) score serves as a running example throughout this paper to illustrate the key concepts and functionalities of the TOP Framework.

A crucial aspect of the TOP Framework’s development is its usability and the experience it offers to researchers and clinicians. Therefore, following the description of the framework’s architecture and functionalities, this paper presents an evaluation of the user experience through an online survey conducted with potential users. This evaluation aimed to assess the framework’s intuitiveness, efficiency, and overall suitability for supporting phenotyping tasks in real-world settings.

This paper aims to contribute a valuable tool and methodology to the field of phenotyping by detailing the design, implementation and evaluation of the TOP Framework, thereby facilitating a deeper understanding of disease and the advancement of personalised healthcare. The framework is already in use within the MII, exemplified by its application in the POLAR and INTERPOLAR projects for modelling adverse events [16]. Evaluations outside the MII include various analyses based on data from the large epidemiological study LIFE [17].

## Implementation

### Theoretical foundation

This section introduces the core notions underlying the TOP Framework. All essential elements and functionalities of the framework will be illustrated using the MELD score [18] as a running example.

#### MELD score

The MELD score (Model for End-Stage Liver Disease) has originally been developed to predict the three-month mortality rate after implementation of a transjugular intrahepatic portosystemic shunt. Later it was shown that MELD can also be used to predict mortality in patients with liver diseases of different origin and varying severity of disease [19, 20]. The initial MELD score is calculated from the laboratory parameters serum creatinine, bilirubin and International Normalised Ratio (INR) [18]. Based on the MELD, several variants have been developed including additional or excluding specific parameters. Examples are the MELD-Na, which includes serum sodium as a sensitive parameter for liver cirrhosis, or MELD-Xi, excluding the INR from the calculation for patients taking Vitamin-K-antagonists which intrinsically will elevate this parameter [21]. MELD, and later MELD-Na, have been used to allocate organs for liver transplantation. Since July 2023, a new MELD variant has been published for this task which now includes sex recorded at birth (or adjusted according to gender-affirming hormone therapy, if applicable), along with the laboratory parameters bilirubin, serum sodium, INR, albumin, and serum creatinine [22].

Although not meant for patients without liver disease, the MELD and MELD-NA score have been used as general risk prediction tools, for instance for postoperative complications, perioperative risks, length of hospital stay or mortality [23–27]. In addition, MELD and MELD-Na have been studied as screening tools for the identification of patients with liver disease at hospital admission by pharmacists [28, 29]. This is of special interest, because the MELD can be correlated to the Child-Pugh-Score which is recommended as the basis of drug adjustment to liver function by the Food and Drug Administration and the European Medicines Agency [23, 30–32].

Importantly, for correct calculation of the MELD score, correction to standard levels is needed. Laboratory values less than 1.0 have to be set to 1.0 and serum creatinine has to be set 4.0 in patients with levels higher than that, patients who received two or more dialysis treatments or 24 hours of continuous dialysis within the prior seven days according to the OPTN recommendations valid until July 2023 [33]. The new recommendation from July 2023 lowered that to 3.0, respectively. For MELD-Na, serum sodium levels less than 125 mmol/l will be set to 125 and greater than 137 mmol/l set to 137 [22, 33]. Moreover, additional factors interfering with the MELD parameters have to be considered appropriately. This is especially important for drugs elevating the INR including the Vitamin-K-Antagonists and direct acting oral anticoagulants, and diagnosis of renal impairment leading to an elevated serum creatinine level. For bilirubin, presence of Morbus Meulengracht may be of concern [28].

#### Phenotype

Phenotype is a fundamental concept in our TOP Framework, representing any (combination of) bodily feature(s) or observable characteristic(s)/trait(s) of an organism [1, 2]. These can manifest in various forms:

- **Directly Observed:** Such as sex or age.
- **Measured:** Obtained through instruments or assays, including laboratory parameters (e.g., creatinine, bilirubin) or gene variant measurements.
- **Derived:** Calculated or inferred from other phenotypes. A key example throughout this paper is the MELD score, which is derived from laboratory measurements of bilirubin, creatinine, and INR.

Importantly, our definition extends the traditional notion of phenotypes to include **received medications and medical procedures**. Such phenotypes can be interpreted not only as the fact that a patient has taken a particular drug or undergone a particular procedure, but they also provide contextual information about a patient’s condition. For instance, the administration of a particular drug may suggest an underlying condition or symptom, while a medical procedure might indicate a specific diagnosis or treatment pathway. Furthermore, these phenotypes can have downstream effects that manifest as other observable phenotypes (e.g., medication leading to a change in a laboratory value). This information plays a central role within the TOP Framework in drawing conclusions about the patient’s condition and in identifying or deriving further phenotypes.

**Example 1:**Phenotype Set of John Doe

John Doe is a patient with the following phenotypes:

- Age: 35 years
- Bilirubin: 1.8 mg/dL
- Creatinine: 1.4 mg/dL
- International normalised ratio (INR): 1.5
- Oral anticoagulant intake: No
- Dialysis status: No
- MELD score: 16.4

#### Phenotype class

Building upon the definition of a phenotype as an observable characteristic or trait, we now introduce the concept of **phenotype classes**. From an ontological perspective, a differentiation is made between individuals (uniquely determined entities which cannot be instantiated) and categories (instantiable entities which can be predicated of other entities) [34]. Applying these notions to our approach results in the following definition: phenotype classes are categories whose instances are phenotypes. For example, the phenotype class ‘creatinine’ is instantiated by specific creatinine concentrations in serum of humans.

Furthermore, we define a **phenotype restriction** as a phenotype class whose extension (the set of instances, i.e., the phenotypes belonging to that class) is limited to a specific value range or condition. For instance, ‘MELD score ≥ 15’ is a phenotype restriction derived from the phenotype class ‘MELD score’.

**Example 2:** Phenotype Classes

- **Phenotype classes:** Age, bilirubin, creatinine, INR, intake of oral anticoagulants, dialysis status, MELD score
- **Phenotype restrictions:** MELD score ≥ 15, age ≥ 12 years, intake of oral anticoagulants = Yes

#### Phenotype Model

A **phenotype mode**l is a formal representation of delimited phenotypic domain knowledge by describing phenotype classes, their attributes, and relationships [15]. The modeller can specify a phenotype class as either basic or derived, depending on the use case. Basic phenotype classes (originally named ‘single phenotype classes’) represent phenotypes that have already been determined and for which the data are available. In contrast, derived phenotype classes (originally named ‘composite phenotype classes’) model phenotypes that should be deduced from other phenotypes and thus require a derivation rule to be formulated. A derivation rule is a calculation instruction that may be represented by a simple mathematical or Boolean formula or may involve complex functions or require extensive computation.

A detailed classification of phenotypes is provided in the Core Ontology of Phenotypes [35]. Originally, the terms ‘single’ and ‘composite’ phenotype class were used. However, we decided to rename these to ‘basic’ and ‘derived’ phenotype class, respectively. This change better reflects the fact that instances of basic phenotype classes can be based on multiple phenotypes. For instance, if the data source already includes the body mass index (BMI) value, there is no need to model the corresponding derivation rule (from weight and height). Consequently, BMI can be modelled as a basic phenotype class. On the other hand, derived phenotypes do not necessarily depend on multiple phenotypes but can also be inherited from only one phenotype (e.g., adjusted bilirubin value in the MELD model). A modified version of the Core Ontology of Phenotypes is available at [36].

**Example 3:** MELD Phenotype Model

The MELD model provides a formal description of all necessary knowledge required for calculating and classifying the MELD^1^ score. This encompasses all required phenotype classes and their attributes, including title, data type, unit of measure, and codes from established terminologies. Additionally, the MELD score formula (derivation rule) [18]

$$\begin{aligned}\text{MELD score} &= 9.57 \cdot ln\left( {Creatinine} \right) + 3.78 \cdot ln\left( {Bilirubin} \right)\cr&\quad + 11.2 \cdot ln\left( {INR} \right) + 6.43\end{aligned}$$

and value ranges (phenotype restrictions) are included. Furthermore, rules for the adjustment of some parameters are defined. If the values for bilirubin, creatinine, or INR are less than 1, 1 must be used instead in the calculation. However, if creatinine is greater than 4 or the patient received at least one continuous (24 hours) or two intermittent dialysis treatments within 7 days prior to the creatinine measurement, the value must be set to 4.

The whole MELD model was developed with the TOP Framework, is accessible at [37], and can be downloaded in a vendor-neutral JSON format.

#### Phenotype query

A **phenotype query** is a specification of certain phenotype classes (or their restrictions) as inclusion or exclusion criteria. The purpose of a phenotype query is to identify individuals whose phenotypes satisfy all the inclusion criteria and none of the exclusion criteria. More precisely, it aims to select individuals who exhibit phenotypes that are instances of the specified inclusion phenotype classes (or fall within the defined restrictions) and do not exhibit phenotypes that are instances of the exclusion phenotype classes (or fall within their restrictions). A preliminary classification of phenotype queries has been proposed in [15].

**Example 4:** MELD Phenotype Query

This query aims to identify individuals who are likely candidates for MELD score evaluation based on certain criteria:

- **Inclusion criterion:** Age ≥ 12 years
- **Inclusion criterion:** MELD score ≥ 15
- **Exclusion criterion:** Intake of oral anticoagulants

#### Phenotype algorithm

In general, an algorithm is a clear specification of instructions to solve a problem or a class of problems [38]. Static algorithms solve a specific, single problem, whilst dynamic algorithms are capable of solving a class of problems. Within the realm of phenotyping, a phenotype algorithm is commonly understood as an algorithm intended to identify or classify phenotypic traits [39, 40]. In other words, the algorithm identifies individuals who match a specific or arbitrary phenotype query. We presented an initial methodology on simple dynamic phenotype algorithms in [41]. This methodology has been expanded and incorporated into the TOP Framework and is outlined below.

Phenotype models and queries can be formally specified using the TOP Framework. The TOP Framework implements a dynamic phenotype algorithm that takes as input a formal specification of an arbitrary phenotype query. Such queries are constructed based on the criteria and logic defined within a corresponding phenotype model. This dynamic nature allows the TOP Framework to be applied to a wide range of phenotyping tasks by simply providing different phenotype query specifications. Currently, the algorithm supports relational databases and Health Level 7 Fast Healthcare Interoperability Resource (HL7 FHIR) [42] servers as data sources that can be queried for individuals using SQL and HL7 FHIR Search [43], respectively. The algorithm searches for individuals matching the criteria, always performing the same core steps:

- **Generate and execute queries for basic phenotypes:** The algorithm generates and executes queries (SQL or HL7 FHIR Search) to retrieve data for the basic phenotypes specified in the query.
- **Derive additional properties (derived phenotypes):** Based on the retrieved data, the algorithm derives additional phenotypes as defined in the phenotype model and query. For example, for a MELD score query, this step involves calculating the score using the retrieved values for bilirubin, creatinine, and international normalised ratio (INR).
- **Check inclusion/exclusion criteria:** The algorithm evaluates the retrieved and derived phenotypes for each individual against the inclusion and exclusion criteria defined in the phenotype query.
- **Provide results:** Finally, the algorithm outputs a list of individuals who satisfy all inclusion criteria and none of the exclusion criteria. This output is provided in both machine-readable format (CSV) for further processing and human-readable format (tables) for easy interpretation.

### Online survey on the user experience of the Framework

To evaluate the overall user experience of the TOP Framework, an anonymous online survey was conducted. The survey aimed to assess various aspects of the framework, including the comprehensibility of the user interface and underlying functionalities, visual design, trustworthiness, and perceived novelty and utility. No personal data about the participants were collected. Implied informed consent was obtained from all participants, as submission of the anonymous survey was considered an indication of consent after reviewing a clear description of the study purpose.

For the survey we used the User Experience Questionnaire UEQ (version 11) [44, 45], a standardised questionnaire for measuring user experience. The questionnaire can be used to compare two software products or to evaluate a sole product for sufficient usability and to identify areas for improvement. It consists of 26 items from 6 scales (attractiveness, perspicuity, efficiency, dependability, stimulation, and novelty) without collecting personal data. All 26 items are implemented as scales from −3 to 3 (seven options to choose from), where participants have to decide between two opposite attributes (e.g., attractive and unattractive), where −3 is the most negative, 0 is neutral, and 3 is the most positive answer. When interpreting the scale, it is important to note that values between −0.8 and 0.8 represent a neutral evaluation, while values above 0.8 represent a positive evaluation and values below −0.8 represent a negative evaluation. The authors also point out that it is extremely unlikely that values below −2 or above 2 will be observed, as means are calculated across people with different opinions and response tendencies. Analysis of the results were performed with R version 4.3.3 [46].

Survey responses were collected and managed anonymously using REDCap electronic data capture tools hosted at the Leipzig University [47, 48]. REDCap (Research Electronic Data Capture) is a secure, web-based software platform designed to support data capture for research studies, providing 1) an intuitive interface for validated data capture; 2) audit trails for tracking data manipulation and export procedures; 3) automated export procedures for seamless data downloads to common statistical packages; and 4) procedures for data integration and interoperability with external sources.

The anonymous online survey was distributed in 2024 via a public link promoted on the TOP Framework homepage and during two related workshops. Additionally, the survey link was disseminated to all colleagues within the SMITH consortium. This resulted in a heterogeneous study population comprising computer scientists, medical staff, pharmacologists, and research assistants from diverse disciplines. Participants’ experience with the TOP Framework varied, with some having prior experience and others being introduced to it during the workshops. There were no significant changes to the framework in the time of survey distribution that may have influenced the results.

### Implementation of the TOP Framework

This section describes the TOP Framework and especially its core module, the Phenotype Reasoner.

#### The TOP Framework

**Fig. 1:**
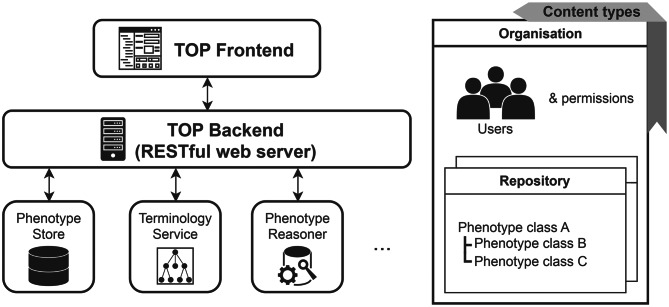
An overview of the entire TOP Framework and its multiple modules (e.g., phenotype store, phenotype Service, phenotype Reasoner) connected to the backend. The TOP API, represented by arrows, mediates communication between backend and the different modules. The hierarchy of content types is shown on the right side

The aim of the TOP Framework is to support the creation, maintenance, dissemination, and implementation of phenotype models. It comprises several individual modules, each designed for a specific purpose. The framework’s domain model has been integrated into the TOP API [49], an OpenAPI-based specification of communication endpoints, message formats, and content types. A RESTful web server, called TOP Backend [50], bundles all modules, with communication between the backend and modules facilitated by the TOP API (see Fig. 1). All features can be programmatically accessed through the TOP API or via the TOP Frontend [51], which serves as a user-friendly web application accessible from any internet browser. The core phenotyping logic, responsible for processing phenotype queries, resides within the Phenotype Reasoner module. The architecture and functionalities of these modules are detailed in the subsequent sections.

Within the framework, we distinguish between content types for organisations, repositories, and phenotype classes. Furthermore, there is a content type for users that is applicable when the framework is configured to necessitate authenticated requests. Users may obtain authorisations to read or modify the content of one or more organisations. Organisations themselves can include repositories that act as a compilation of phenotype classes.

**Fig. 2:**
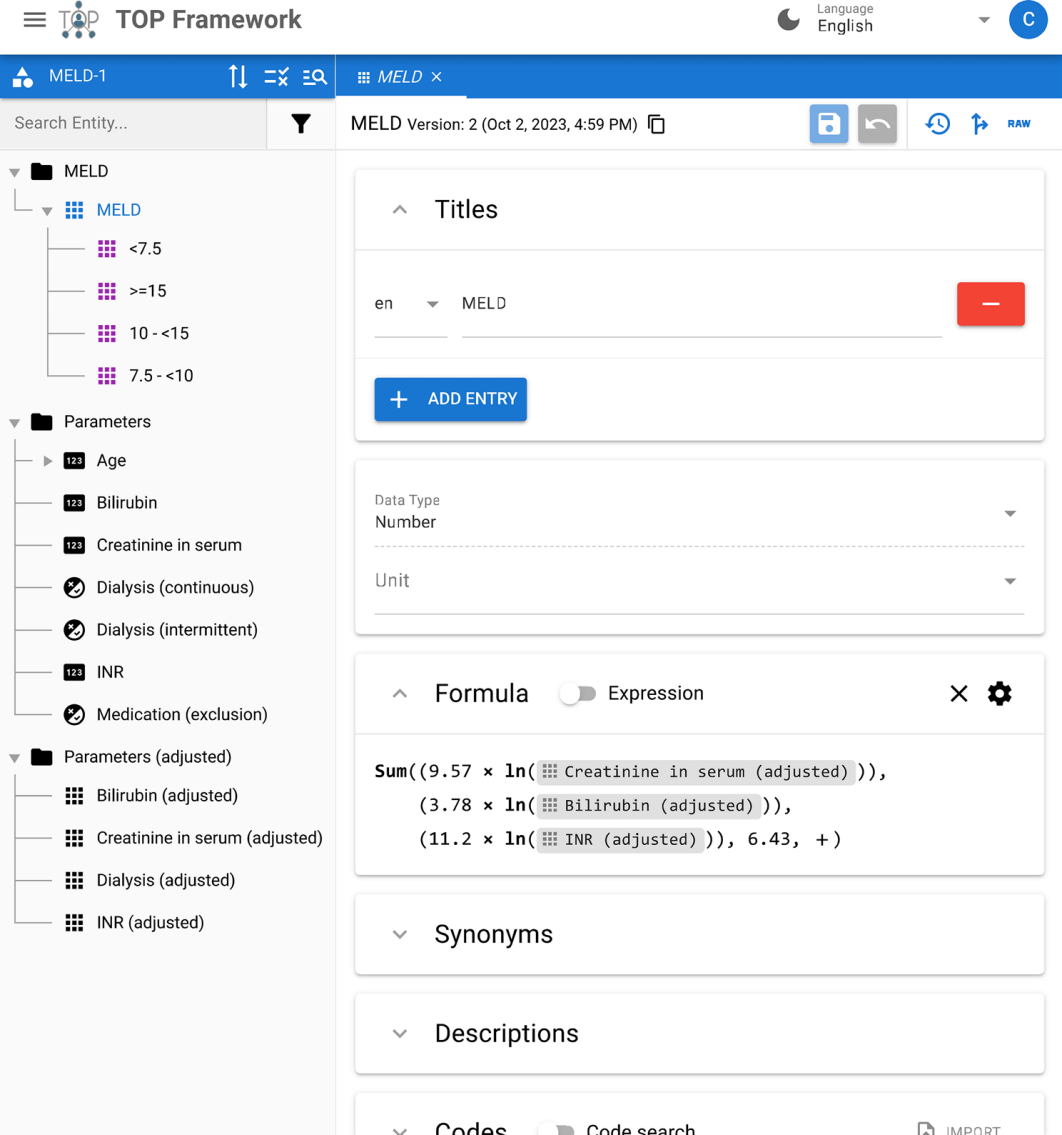
Screenshot of the phenotype editor component of the TOP Framework, showing the ‘MELD’ repository. On the left side, a tree with all phenotype classes is shown and on the right side, properties of the composite phenotype class ‘MELD’ are listed. This screenshot was taken from our public demo instance

The Phenotype Editor, a key component of the TOP Frontend, provides an intuitive interface for creating and manipulating repositories and their contained phenotype classes. As an example, we have created a repository named ‘MELD’ within the TOP Framework. This repository contains all the relevant phenotype definitions for calculating the MELD score. Please refer to Section “User experience evaluation results” for more information about the phenotypes. Figure 2 displays a screenshot of the TOP Framework, which shows the repository in the Phenotype Editor component. On the left-hand side, a hierarchical tree of all phenotype classes is displayed, illustrating the superclass-subclass relations. Phenotype classes can be added, selected, and removed from the tree. The phenotype classes have been categorised based on their parameter type and whether they are included in the actual MELD score calculation. Further details of the selected phenotype classes are displayed on the right-hand side in editor tabs, which contain various widgets to manipulate different properties such as title, data type, expressions (e.g., mathematical formulas), and relations to external code systems. In particular, an interactive and reliable method of constructing expressions from predefined functions and phenotype classes as variables is provided by the expression construction UI component.

All content, including phenotype classes managed through the Phenotype Editor, is persisted in the Phenotype Store. The store acts as an interface to a relational database system. Any changes made to a phenotype class are recorded in the version history, allowing users to view and restore previous versions. The repositories containing phenotype classes can be designated as public. This permits other users to browse and reuse the contained phenotype class definitions they contain. References between phenotype classes always point to the latest version, otherwise updating one class to a new version could introduce unwanted new versions for a substantial proportion of the other classes in the repository.

There are two supported methods of reuse within the framework. The first approach involves creating a fork of a phenotype class in other repositories. The term ‘fork’ here refers to the creation of a copy of the class and an equivalence relationship between the forked version and the original class. Forks have independent version histories, allowing them to be modified separately from their origin. If the original phenotype class receives an update, the new version can optionally be added to the version history of the forked class. The other way to facilitate reuse and interoperability is to export the entire repository in a JSON format compliant with the TOP API. This export can be re-imported into another TOP Framework instance or used in custom software tools.

**Fig. 3:**
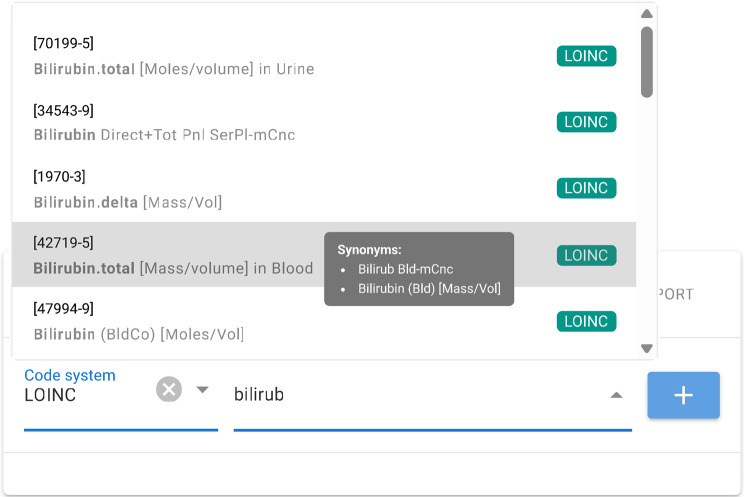
The code auto-completion feature in the TOP Framework frontend reference implementation. The codes and their labels come from the LOINC terminology stored in an OLS instance. This screenshot was taken from our public demo instance

Phenotype classes and concepts managed by the TOP Framework can be mapped to external terminologies and coding systems in order to facilitate interoperability. To this end, the TOP API provides interfaces for integrating external terminology services. The current reference implementation of the TOP Framework contains a connector to the Ontology Lookup Service (OLS, version 4) [52, 53], further implementations can be added. The current implementation provides methods for retrieving terms/codes from various code systems and meta-information about the code system. These can be single codes or even terminology subtrees. The ability to store terminology subtrees simplifies the development of phenotype models, especially when dealing with a large number of related concepts. An auto-completion feature facilitates the mapping procedure for users of the TOP Framework (see Fig. 3). A uniform representation format for codes (which come from highly heterogeneous input sources) is ensured by a declarative import pipeline, which allows mapping of varying input formats (such as CSV, XML, RDF/OWL) and data structures to a common internal format, defined by the TOP Terminology Ontology [54].

Once a TOP entity is mapped to an external code, the code, its universal identifier and its label are stored in the TOP Framework in order to retain the information in case a phenotype class is exported and reimported to another system in which the connection to the terminology service is possibly not available.

The framework is provided as open-source software (MIT licence), which allows IT departments to set up a self-hosted instance, offering complete control over user access permissions and compliance with local data privacy regulations. The software itself and all the required components, including a database and an optional OAuth2-based authentication server, are provided as Docker containers. Please refer to the documentation^2^ for further details.

#### Phenotype reasoner module

**Fig. 4:**
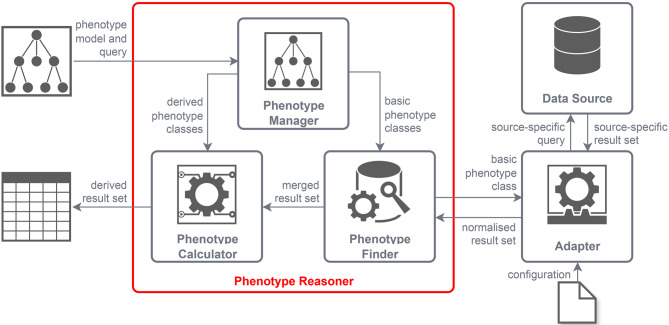
An overview of the phenotype Reasoner components. The arrows indicate the flow of information: phenotype Manager provides the current phenotype query specification to the phenotype Finder, which generates and executes queries (in a specific query language) using a suitable data source adapter, consolidates the requested data and forwards the result set to phenotype Calculator for deriving further phenotypes

The Phenotype Reasoner module is responsible for retrieving required data and deriving further phenotypes. Processing a phenotype query generates a dataset (result set) containing data of individuals (i.e., people) matching the query. Each person in the result set has an individual record, consisting of a list of phenotypes represented by phenotype classes and corresponding values. Internally, the Phenotype Reasoner consists of three core components: Phenotype Manager, Phenotype Finder, and Phenotype Calculator (Fig. 4). Further elaboration on all three core components follows in the subsequent paragraphs.

**Fig. 5:**
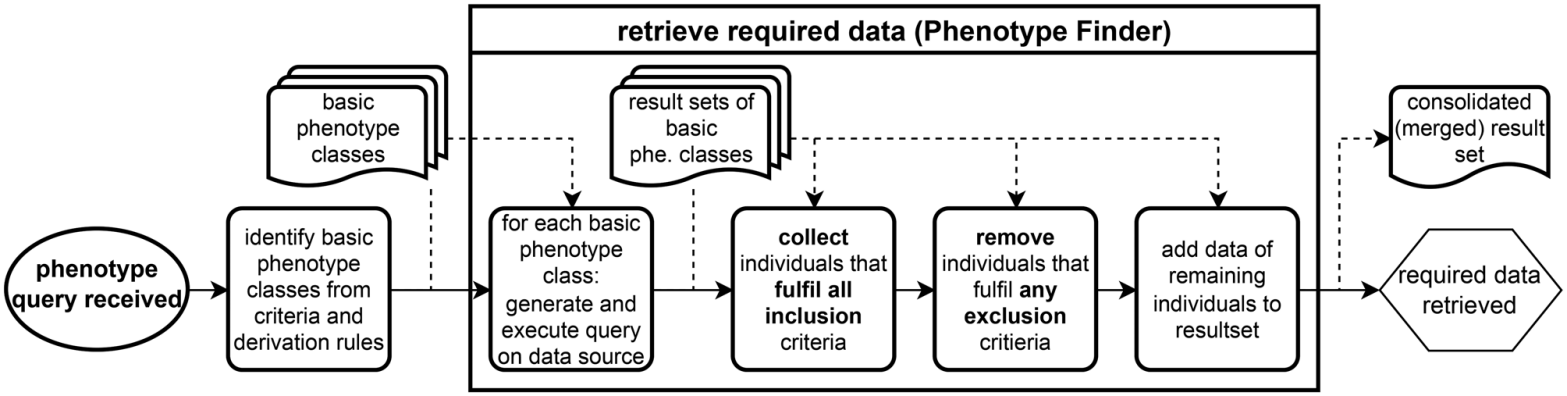
Event-driven process chain of the phenotype Finder component. The initial step is performed by the phenotype Manager

After a phenotype query is initiated in the frontend, it is sent to the Phenotype Reasoner by the backend along with the query specification (inclusion/exclusion criteria and the underlying model). The Phenotype Manager detects all basic phenotype classes, whether they are explicitly mentioned within the inclusion/exclusion criteria or integrated as variables in derivation rules/formulas of derived phenotype classes. The detected classes are transferred to the Phenotype Finder (see Fig. 5). For each basic phenotype class, Phenotype Finder generates and executes a query in a specific query language using a data source adapter. Two adapters for FHIR Search and SQL are incorporated into the system. Each adapter is adjustable by a configuration file that includes connection details for the corresponding data source, query templates, as well as optional mappings for value ranges and units of measurement. Examples of configuration files with accompanying descriptions are given in our data adapter documentation^3^. Query templates may include placeholders enclosed in curly brackets, which are automatically replaced at runtime with the data defined in the corresponding phenotype classes, particularly terminology codes and value ranges. For example, the base query template for FHIR resources of type ‘Observation’ contains a placeholder labelled ‘{codes}’:

*baseQuery: “Observation?code={codes}&_count = 100”*.

To generate an executable query, the placeholder is replaced by codes specified in the corresponding phenotype class. For instance, the query for creatinine is generated as follows: ‘Observation?code=http://loinc.org|2160-0…’. Value ranges in the query are specified through additional query parts such as ‘numberValueIntervalPart’:

*numberValueIntervalPart: “&value-quantity{operator}{value}”*.

The ‘output’ section outlines FHIRPath expressions for retrieving pertinent data such as the subject ID or resource value from the requested FHIR resources:

*subject: subject.reference.value*

*numberValue: value.value.*

The adapter normalises the source-specific result sets (i.e., FHIR bundles or SQL result sets) to TOP Framework’s class model. Following the execution of all basic phenotype queries, Phenotype Finder merges the single result sets. Result sets arising from inclusion criteria are set-theoretically intersected, with only individuals fulfilling all inclusion criteria remaining in the result set. The result sets for exclusion criteria are deducted from the overall result set using set-theoretical difference. All basic phenotype values are added to the corresponding individual records in the overall result set.

**Example 5:** Collection of Initial Records

In the MELD example, individuals aged at least 12 years (inclusion criterion) form the initial result set. People taking oral anticoagulants are excluded from this set (exclusion criterion). Basic phenotype values (creatinine, bilirubin, INR, age, etc.) are added to the individual records.

The consolidated (merged) result set is transferred to the Phenotype Calculator, which derives phenotypes for every person (i.e., individual record) in the set. A recursive approach is used for phenotype calculations, utilising expressions (representation of derivation rules) specified as attributes of derived phenotype classes. Each derived phenotype class requires an expression with a matching data type, including number, text, Boolean, or date-time. These expressions represent one of the following:

- a phenotype class
- a constant/value or a list of constants/values
- a function with a set of arguments (which are also expressions)

For example, a number expression is either a numeric phenotype class (e.g., creatinine or bilirubin), a numeric constant/value (list) (e.g., π or (1, 2, 3)), or a function returning a number expression. A function converts its arguments into a single expression of a defined data type. We implemented a number of common mathematical, Boolean, date-time, aggregate, comparison, and advanced functions that encompass control functions such as ‘If’, ‘Switch’, and ‘ForEach’ as well as set functions such as ‘Filter’, ‘Exists’, and ‘Union’. These advanced functions are the result of a close collaboration with domain experts. Their aim is to offer complex yet practical features in a user-friendly manner. The full documentation of all implemented functions is available at [55]. Further functions can be implemented using a plug-in mechanism.

**Example 6:** Functions in the TOP Framework

The rule for adjusting bilirubin value (if bilirubin is less than 1, use 1) can be modelled using ‘Max’ or ‘If’ function:

$$Bilirubi{n_{adjusted}} = If\left(Bilirubin < 1,1,Bilirubin\right)$$

The following expression is used to check whether dialysis treatments were performed within the 7 days prior to the creatinine measurement:

$$\begin{aligned}Dialysi{s_{adjusted}} =Or( & Exists\left(Filter\left(Dialysis_{continuous}, 7, Creatinine\right)\right), \cr & Count\left(Filter\left(Dialysis_{intermittent}, 7, Creatinine\right)\right) \geq 2)\end{aligned}$$

The ‘Filter’ function reduces the dialysis treatments to those of the last 7 days before the creatinine measurement. The ‘Exists’ function checks whether at least one dialysis remains, while the ‘Count’ function counts the dialysis treatments in the filtered set. The overarching ‘Or’ expression checks whether one of the two conditions is fulfilled, i.e., at least one continuous or two intermittent dialysis treatments exist within the 7 days prior to the creatinine measurement.

The creatinine adjustment rule can be modelled using the ‘Switch’ function, which combines an arbitrary number of if-then pairs and an optional else-expression. If one of the if-expressions is true, the corresponding then-expression is returned, otherwise the else-expression is returned.

$$\begin{aligned}Creatinin{e_{adjusted}} = Switch(& Creatinine > 4, 4,\\ &Dialysis_{adjusted}, 4,\\ &Creatinine < 1, 1,\\ &Creatinine)\end{aligned}$$

The resulting MELD expression is:

$$\begin{aligned}Sum( & 9.57 \cdot ln\left(Creatinine_{adjusted}\right), \cr & 3.78 \cdot ln\left(Bilirubin_{adjusted}\right), \cr & 11.2 \cdot ln\left(INR_{adjusted}\right), \cr & 6.43)\end{aligned}$$

**Fig. 6:**
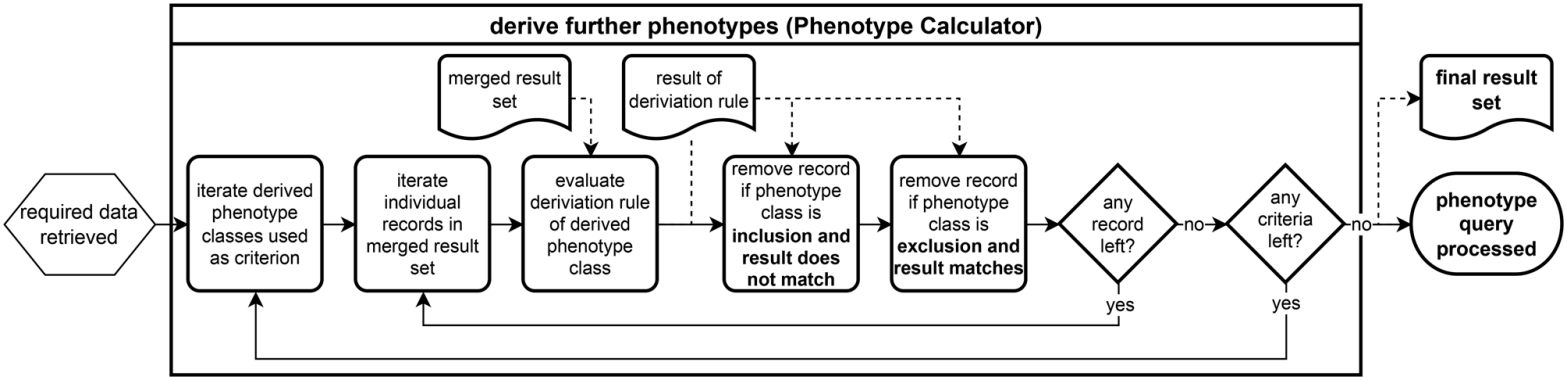
Event-driven process chain of the phenotype Calculator component

The starting point for the Phenotype Calculator is a derived phenotype class, e.g., defined as an inclusion criterion (see Fig. 6). The expression of the class is then processed, with recursive calculations performed for any contained functions. If the expression contains a constant, such as e, π, NOW, or TRUE, it will be replaced by its corresponding value. If another phenotype class is included as a variable, the Phenotype Calculator first checks whether the individual record already contains values for that variable. If not, the phenotype class expression is calculated before proceeding.

If a derived phenotype is an instance of the inclusion criteria class or is no instance of the exclusion criteria class, the corresponding individual record remains in the result set and the derived phenotype value is added. Otherwise, the corresponding record is excluded from the result set.

**Example 7:** Derived Phenotype as an Instance of an Inclusion Criterion

The MELD score is calculated for all patients. The expression ‘MELD > 15’ (inclusion criterion) serves as an entry point. It contains a function (operator ‘>’) that returns a Boolean value. Since the MELD value is not yet contained in the individual record, it must be calculated. The MELD expression contains three (adjusted) variables for which expressions also exist. The adjustment expressions are based on the original values contained in the individual record and can therefore be calculated. The MELD value is then recursively processed. Patients with MELD over 15 remain in the result set, the others are excluded.

#### Applications of advanced functions

This section aims to demonstrate the breadth of the function library by means of additional examples.

In order to identify cases of Type 2 Diabetes Mellitus (T2DM) [56], it is necessary, amongst other things, to check whether the first administration of a T2DM medication preceded the first administration of a T1DM medication. In the TOP Framework, this can be modelled as follows:

$$StartsBefore\left(\begin{aligned} {First\left( {T2DMmedication} \right), First\left( {T1DMmedication} \right)} \end{aligned}\right)$$

The ‘StartsBefore’ function returns ‘true’ if the process represented by the first argument starts before the process represented by the second argument; otherwise, it returns ‘false’. In our case, the function is applied to the first T2DM and T1DM medication administrations.

As part of the Acute Kidney Injury (AKI) algorithm [57], the current serum creatinine level is compared to the lowest value measured within the 7 days prior to the current measurement. To determine this value, the TOP Framework uses the following expression:

$$Min\left( {refValues\left( {Creatinine,7} \right)} \right)$$

The ‘RefValues’ function reduces the number of elements in a set (the values of the first argument, i.e., all creatinine values) by applying a date range restriction. The second and optional third arguments specify the date range in days relative to the most recent value (index), e.g., 2–4 or 7 days before the index value. Only those values measured within the defined interval remain in the set. The ‘Min’ function applied to this set returns the lowest value.

One of the criteria for identifying patients with delirium [16] is that at least two of the following three factors must be present: age over 80, infection, and a sodium level outside the defined range:

$$\begin{aligned}Min\# true( & 2,\\ & \text{Age: Above 80 years},\\ & Infection,\\ & Or\left(\text{Sodium level: < 135mmol/L}, \text{Sodium level: > 145 mmol/L}\right))\end{aligned}$$

The first argument of the ‘MinTrue’ function specifies the minimum number of subsequent arguments that must be true. If at least that many arguments are true (starting with the second argument), the function returns ‘true’; otherwise, it returns ‘false’.

#### Automated model testing

To facilitate the validation and verification of developed phenotype models, the TOP Framework incorporates a dedicated testing module. This feature empowers users to automatically assess the correctness of their models against custom test datasets.

The framework allows users to upload their own test data in a standardised format (CSV). This flexibility ensures that users can evaluate their models using specific data relevant to their context. Upon uploading the test data, users can initiate the testing process through the framework’s interface. The TOP Framework then automatically executes the developed phenotype model against the provided test dataset. This process involves generating phenotype queries and applying the model’s logic and parameters to the test data to derive phenotype values.

**Fig. 7:**
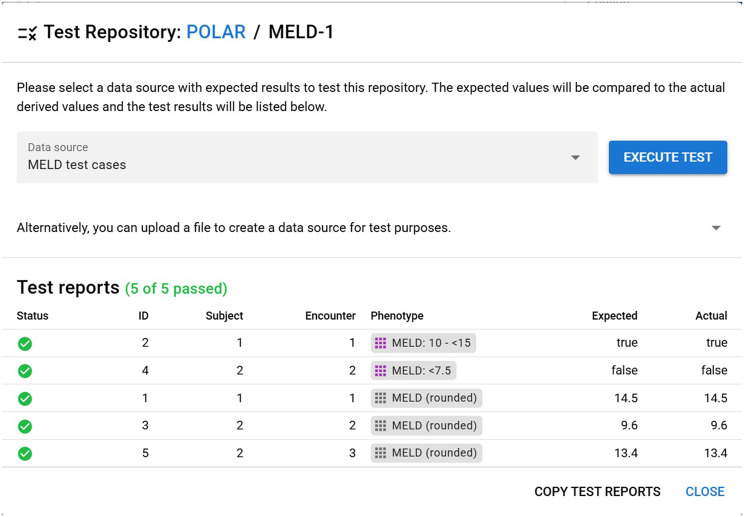
This figure shows a simplified screenshot of the testing module within the TOP Framework. For simplicity reasons the derived phenotype class ‘MELD (rounded)’ was used, where the MELD score is rounded to one decimal place. The screenshot was taken from our public demo instance. However, access to repository tests is restricted to registered users with appropriate permissions. The module displays test reports for the MELD score, derived from synthetic data. Each row in the table corresponds to a single test, where an expected value was predefined. The phenotype Reasoner module was used to query the underlying test database and compute the actual values. The “status” column indicates the outcome of each test, with green checkmarks signifying successful results. The test data set that was used to take this screenshot is available as CSV files in [58]

Following the execution, the framework generates a comprehensive evaluation report. This report includes a list of expected values, as defined in the test dataset, and the actual derived values, as well as the result of the comparison between the two (see Fig. 7). The generated evaluation report provides users with immediate feedback on their model’s correctness. The ability to easily assess and refine models within the framework promotes a more robust and reliable phenotype modelling process.

## Results

In the following sections, the results of the usability evaluation are presented and a comparison of the framework’s functionalities to the important desiderata of Mo et al. and Chapman et al. is given.

### User experience evaluation results

In this work, we focused on a preliminary usability evaluation of the TOP Framework and conducted the UEQ for this purpose. The survey was distributed in 2024 to computer scientists, medical staff, pharmacologists, and research assistants, who had either already actively used the TOP Framework or had gained initial experience as part of our workshops. We received 11 responses. While one response was incomplete due to a missing answer on the dependability scale (item about whether it meets expectations or not), we decided to include the remaining data in our analysis as it still provided sufficient information for the other scales.

The UEQ assesses user experience across three higher-order scales: attractiveness, pragmatic quality (*perspicuity*, *efficiency*, and *dependability*), and hedonic quality (*stimulation* and *novelty*). The results for the TOP Framework indicate a generally positive user experience. Specifically, the framework received a mean score of 1.50 (CI = (1.07, 1.93)) for attractiveness, suggesting users find it visually appealing and likeable. The pragmatic quality scale achieved a mean score of 1.38 (CI = (1.00, 1.77)), indicating users perceive the framework as clear, efficient, and reliable. Finally, the hedonic quality scale resulted in a mean score of 1.40 (CI = (0.76, 2.03)), suggesting users find the framework engaging and interesting to use.

Further analysis of the individual UEQ scales (see Table 1) reveals that all mean scores are above 0.8, indicating a positive perception across all aspects of usability. Notably, the stimulation (1.70, CI = (1.20, 2.21)) and attractiveness scores were particularly high, suggesting the user interface is perceived as clear, appropriate, and engaging. This is likely supported by the explicit communication of functionality through tooltips and introductory text provided for all graphical elements and fields, ensuring users understand the purpose of each component. Furthermore, the absence of obscure design patterns facilitates smooth navigation within the application, preventing user confusion. The dependability scale also stands out with a mean score of 1.51 (CI = [0.99, 2.03]), indicating a strong perception of trustworthiness and freedom from errors or data loss among users.

**Table 1:** Individual results of all scales of the UEQ

	Responses, *N* = 11				
Scale	Mean (SD)	Median (Q1, Q3)	Min - Max	95% CI	Interpretation
attractiveness	1.50 (0.64)	1.33 (1.17, 2.00)	0.50 - 2.50	(1.07, 1.93)	positive
perspicuity	1.20 (0.73)	1.25 (0.75, 1.75)	−0.25 - 2.25	(0.71, 1.70)	neutral - positive
efficiency	1.43 (0.75)	1.50 (0.75, 2.25)	0.25 - 2.25	(0.93, 1.94)	positive
dependability	1.51 (0.78)	1.25 (0.75, 2.25)	0.33 - 2.50	(0.99, 2.03)	positive
stimulation	1.70 (0.76)	2.00 (1.50, 2.25)	0.00 - 2.50	(1.20, 2.21)	positive
novelty	1.09 (1.20)	1.50 (−0.25, 2.00)	−0.75 - 2.75	(0.29, 1.89)	neutral - positive

Given are means with standard deviation (SD), medians with 25% (Q1) and 75% (Q3) quantiles, minimum, maximum, and 95% confidence intervals (CI). Values between −0.8 and 0.8 represent a neutral evaluation, while values above 0.8 represent a positive evaluation and values below −0.8 represent a negative evaluation. Interpretations derived based on whether the 95% CI fell below, between, or above the cut-off values −0.8 and 0.8, or overlapped them.

### Notes about query performance

The TOP Framework has been subjected to extensive testing to ensure both its functional accuracy and performance efficiency. Unit tests have been conducted on the majority of its functionalities to guarantee reliable and correct outcomes. Our evaluations demonstrate that the framework delivers accurate results within practical timeframes. While the asynchronous nature of phenotypic queries mitigates the need for immediate results, the observed execution times are nonetheless efficient. For instance, a query executed on our demo instance (Intel(R) Xeon(R) Silver 4216 CPU @ 4 × 2.10 GHz, 48GB RAM) with a restriction of MELD score < 7.5 completes in approximately 17 seconds against a PostgreSQL 16 database with synthetic data of 10.000 patients. However, query execution times heavily depend on the data source system, queries are run against and the query complexity (e.g., queries involving multiple complex inclusion/exclusion criteria or requiring data from several phenotype classes). Specifically, FHIR queries tend to be much slower than SQL queries (by a factor of between 10 and 50) due to the less efficient FHIR Search API. These results suggest that the TOP Framework can effectively support a wide range of phenotypic analyses without introducing substantial overhead, even when dealing with large patient cohorts.

We believe this performance is suitable for the intended use cases. Future work may explore further optimisations for specific query types or data distributions to potentially reduce execution times even further. The observed difference in execution times between SQL and FHIR queries is largely due to the distinct data retrieval paradigms employed by each. FHIR Search, as utilised by the Phenotype Reasoner Module (specifically the Phenotype Finder component), often involves querying data for basic phenotype classes through separate requests. In contrast, SQL allows for the retrieval of data from multiple classes within a single query, even with intricate filtering conditions. These performance characteristics suggest that while both SQL and FHIR are valuable for the TOP Framework, SQL currently offers faster retrieval for complex phenotype models involving multiple phenotype classes. FHIR Search, on the other hand, provides interoperability and adherence to the FHIR standard, which are crucial for the integration of the framework in the MII.

### Comparison to desiderata for computable phenotypes and phenotype libraries

To assess the TOP Framework’s alignment with established standards, this section contrasts its features against the desiderata proposed by Mo et al. for computable phenotypes [7] (Table 2) and by Chapman et al. for phenotype definitions and libraries [11] (Table 3).

**Table 2:** Important requirements for computable phenotypes proposed by Mo et al.

Requirement	Status	Solution in TOP Framework
Support both human-readable and computable representations	supported	HTML representation in TOP Frontend, TOP API JSON format as computable representation, export to other formats possible
Implement set operations and relational algebra	supported	Realised in derived phenotypes
Represent phenotype criteria with structured rules (nested structures, Boolean, comparators, aggregation, negation)	supported	Realised in derived phenotypes
Support defining temporal relations between events	supported	Realised in derived phenotypes
Use standardized terminologies and code value sets	supported	Terminology information is stored in JSON representation, connection to Terminology Service
Define representations for text searching and natural language processing	partially supported	Handled with a separate component for semantic text search.
Provide interfaces for external software algorithms	supported	TOP API, Plugin mechanic
Maintain backward compatibility (support old EHR data, such as old codes or data structures)	partially supported	It is possible to connect phenotypes to a variety of terminologies, such as ICD-10 and ICD-9. Old data sources can be connected with tailored query adapters.

**Table 3:** Important requirements for phenotype definitions and libraries proposed by Chapman et al.

Requirement	Status	Solution in TOP Framework
Phenotype library with a formal phenotype model that governs the structure and syntax of phenotype definitions	supported	The TOP Framework serves as a phenotype library, the TOP API is the formal phenotype model, and phenotype classes defined in the framework are the actual phenotype definitions.
Phenotype definitions are formalisations of derivation rules	supported	There is the special derived phenotype class for formalisations of derivation rules.
Phenotype definition formats that should be supported:		
Code lists	supported	Codes can be retrieved from an external terminology server and assigned to all phenotype classes.
Simple data elements (use logical connections for code lists)	supported	Phenotype classes can be associated with multiple codes, connected with logic “OR” operators. Additionally, more complex logical connections are supported through derived phenotypes.
Complex data elements (relationship between complex data elements, e.g., numerical or derived via NLP)	partially supported	NLP and semantic search in text documents is partly supported.
Temporal	supported	Realised in derived phenotypes
Trained classifier	unsupported	The TOP Framework supports rule-based phenotypes. Classifiers are currently not supported.
Implementation of a phenotype definition should be executable and directly runnable against a patient dataset to derive cohorts with defined phenotype.	supported	Realised in Phenotype Reasoner module
Modelling language that is used to define phenotypes. The language should not be a low-level programming language that is simultaneously used to implement an executable version. It should be a precise and clear high-level modelling language.	supported	A proprietary JSON format is used to define phenotypes.
High-level graphical representation of the phenotype definition.	supported	Phenotype definitions, along with metadata, are displayed graphically in the TOP Framework user interface. Simple phenotype models can be exported to the Decision Model and Notation (DMN) format, which in turn can be viewed using appropriate DMN editors.
Support Natural Language Processing-based and machine learning-based definitions.	partially supported	Handled with a separate component for semantic text search.
Complex rules and clinical terminology contained in the definition should be described on multiple abstraction levels to improve clarity.	supported	Comments can be provided as additional metadata of a phenotype class.
Versioning and data provenance, where all versions have a unique identifier.	supported	The version history of a phenotype class is preserved with some provenance data. All versions are accessible via an identifier.
Modular relationships between phenotype definitions to construct new definitions.	supported	Phenotype definitions can be reused in other definitions.
Communicate implementation information in the model.	not applicable	Implementation is realised through a generic algorithm that can be used as it is without the need for further implementation.
Support tooling that provides multiple programming language implementations.	partially supported	Phenotype queries can be performed in any kind of query language. There is currently only a Java implementation available for the Phenotype Reasoner.
Support tooling that provides connectivity with multiple data standards.	supported	The TOP Framework can be connected to multiple data standards using configurable query adapters.
Validation	partially supported	Plausibility checks are possible by providing simple test cases. This is not equal to a thorough validation.
Sharing of definitions (including comprehensive metadata), use of a standard API to access functionalities of the library, and advanced search capabilities.	supported	Data sharing and API access to functionalities and search are supported by the framework.

Both sets of criteria emphasise the necessity for text search capabilities and natural language processing (NLP) support. The TOP Framework includes a component designed for semantic searches within text documents [59], although this functionality is not addressed in the current work. At present, the framework handles queries for structured EHR data and unstructured text data, such as medical reports, through separate processes. Future work will focus on integrating these methods more cohesively. Consequently, the requirements for text search and NLP are considered partially supported.

## Discussion

The TOP Framework presents a novel approach to phenotyping, offering a flexible and efficient solution for defining and querying patient cohorts. A key focus of our evaluation was the user experience, which yielded positive feedback, indicating that the framework is intuitive and user-friendly. This positive reception suggests that the TOP Framework has the potential to be readily adopted by researchers and clinicians. The framework’s ability to handle complex queries across large datasets further highlights its potential for real-world application in medical research. This work contributes to the growing field of computational phenotyping and opens several avenues for future development and research, as discussed below.

Comprehensive and reusable phenotype definitions rely heavily on accurate terminology connections. The TOP Framework aims to improve upon existing solutions by providing enhanced capabilities for navigating and utilising medical terminologies. This includes the ability to specifically target subtrees within a terminology hierarchy and identify leaf nodes, allowing for precise and flexible phenotype definitions. This enhanced terminology connection contributes to a more intuitive and user-friendly experience for researchers defining cohorts. By providing clear and structured access to medical concepts, the framework simplifies the often-complex process of translating clinical knowledge into computable phenotypes.

The ability to reuse existing, validated phenotype definitions and models is crucial for accelerating research and promoting consistency. The TOP Framework currently supports this in two ways: it enables local reuse within the same TOP Framework instance, allowing researchers to readily transfer and apply phenotype definitions across different local models. Additionally, the framework facilitates reuse through a straightforward JSON-based export and import module, enabling the exchange of these definitions and models between different instances. However, this approach is largely limited to direct, instance-to-instance transfers and does not inherently facilitate broader, cloud-based sharing and reuse across distributed research networks.

To address this limitation and significantly enhance reusability, future work should prioritise enabling the seamless integration of phenotype definitions from cloud-based platforms within the TOP Framework. The potential for integrating external plugins, such as the i2b2 plugin described by Visweswaran et al. (2023) [60], offers a compelling example of how cloud infrastructure can fundamentally improve the reuse of phenotype definitions. This particular i2b2 plugin leverages a cloud-based searchable repository, demonstrating an approach for enabling investigators to reuse phenotypes across sites, thereby drastically reducing the need for users to manually search across disparate instances or to construct definitions de novo.

Inspired by such successful examples, a promising avenue for the TOP Framework involves the development of a centralised platform specifically designed to browse phenotype definitions hosted within registered TOP Framework instances. This centralised repository would function as a hub, dynamically linking to the actual TOP instances where the definitions reside. Alternatively, leveraging existing, widely adopted solutions like PheKB [9] presents a compelling strategy. By establishing mechanisms to refer to TOP instances holding specific phenotype definitions from within platforms like PheKB, we could effectively promote and disseminate TOP Framework-based definitions to a broader research community. This approach would capitalise on established infrastructure for phenotype curation and sharing, fostering greater interoperability and collaborative development.

The adoption of common data models (CDMs) like the OHDSI OMOP model, as exemplified by the eMERGE network [61], is essential for facilitating data integration and collaboration. Within the TOP Framework, we already have successfully established a connection to the OMOP CDM. Future exploration of deeper integration with CDMs would not only further extend the TOP Framework’s utility but also simplify the process of working with diverse datasets for users familiar with these models. This alignment could contribute to a more seamless and integrated user experience for researchers working across different institutions and data sources.

A significant future direction involves the integration of the TOP Framework with clinical decision support systems. This would enable the automated provision of alerts or recommendations based on defined phenotypes directly within clinical workflows. The underlying Phenotype Reasoner Module of the TOP Framework could be developed into a standalone component or integrated as a plugin into other software, allowing it to load phenotype models and provide real-time reasoning capabilities to support clinical decision-making.

While the TOP Framework already offers data adapters for various data sources, the development of a comprehensive plugin system is envisioned. This would extend beyond data access, enabling the integration of new reasoning functionalities, advanced analytical tools, and even interfaces to external machine learning models for more sophisticated phenotyping.

The Phenotype Execution and Modelling Architecture (PheMA) [62] is another notable system for defining and executing phenotypes against FHIR or OHDSI OMOP services. While initially promising, development of PheMA ceased in 2021, and it has since remained unmaintained. This lack of updates not only limits its compatibility with evolving data standards but also exposes it to potential security risks, reducing its long-term viability for clinical and research applications.

PheMA employs the Clinical Quality Language (CQL) for phenotype definitions, which avoids introducing a new modelling language, which is a clear advantage. However, CQL is a highly complex language, requiring significant input from informaticians to develop and refine models. This dependency on specialised expertise creates a barrier for clinical users, potentially slowing adoption and limiting the creation of new phenotype models. In contrast, the TOP Framework mitigates this challenge by offering a simplified, user-friendly interface that reduces the technical burden on clinicians and researchers. By making phenotype modelling more accessible, the TOP Framework encourages wider participation in model development.

Future work could explore interoperability between the TOP Framework and legacy systems like PheMA, particularly in leveraging CQL where advantageous while preserving the TOP Framework’s ease of use. Additionally, sustained maintenance and community engagement will be essential to prevent the obsolescence observed in PheMA, ensuring the TOP Framework remains a robust and future-proof solution.

To further establish its value, the TOP Framework will be applied and evaluated across a broader range of clinical domains and research contexts. Through targeted case studies and pilot implementations, we aim to demonstrate its practical utility and tangible benefits in diverse healthcare settings, reinforcing its role as a versatile tool for precision medicine.

### Comparison of the German Portal for medical research data and the TOP Framework

Recently, the German Portal for Medical Research Data (Forschungsdatenportal für Gesundheit, FDPG, a centralised infrastructure for accessing routine health data from university hospitals across Germany [63], was released as a part of the MII. At first glance, it might appear quite similar to the TOP Framework, but there are important distinctions between the two.

The FDPG offers unique advantages, particularly in its accessibility to a broader audience beyond the scientific community. It provides a transparency registry of research projects, making it easier for patients, researchers, and the public to track ongoing and completed studies. Additionally, the FDPG includes a research proposal management module, streamlining the application and approval process for accessing health data.

While both the FDPG and the TOP Framework were developed within the MII, the TOP Framework’s origins trace back even earlier: a first prototype, known as *PhenoMan*, was completed in 2019 [35, 64], well before the FDPG’s public launch in 2022. Unlike the FDPG, which is focused on the MII’s FHIR-based data model, the TOP Framework is not restricted to specific data formats, enabling its application across a wider range of data sources.

Although both platforms enable the identification of patient cohorts based on specific criteria, the TOP Framework emphasises a more theoretical, model-driven approach, allowing users to develop and refine precise phenotype definitions collaboratively. This positions the TOP Framework as a complementary tool to the FDPG, offering expanded capabilities for phenotype modelling in medical research.

### Limitations and future enhancements based on desiderata

The current iteration of the TOP Framework, while robust, also presents opportunities for further enhancement, many of which stem from desiderata of Mo et al. [7] and Chapman et al. [11] (see Section “Comparison to desiderata for computable phenotypes and phenotype libraries”).

A significant limitation of relying solely on structured data for phenotyping is its inherent incompleteness and potential for inaccuracy [6]. Structured data alone often falls short in accurately inferring phenotypes, partly because codes like ICD-9 are primarily for administrative and billing purposes and may not reflect underlying physiology [65]. Furthermore, critical clinical information, such as clinicians’ observations and insights, is frequently documented in unstructured clinical narratives [66]. Therefore, integrating robust natural language processing capabilities into the TOP Framework is crucial for extracting valuable phenotypic information from unstructured clinical text. This significantly enhances the accuracy and completeness of phenotypes. As discussed in Section “Comparison to desiderata for computable phenotypes and phenotype libraries”, the integration of NLP capabilities into the TOP Framework has already been investigated, with initial results published [59].

Ensuring backward compatibility with previous versions of phenotype definitions and framework components will be vital as the TOP Framework evolves. This will prevent disruption for existing users and facilitate seamless updates. While the TOP Framework excels at rule-based phenotyping, future work could explore the integration of trained machine learning classifiers. This would allow for the development of hybrid phenotyping approaches, combining the interpretability of rule-based definitions with the predictive power of machine learning models.

Expanding the Phenotype Reasoner module’s availability across multiple programming language implementations could enable reuse of phenotype models in other software systems to foster wider adoption within different research communities.

Finally, while initial user feedback is positive, the sample size of the user experience evaluation was small (*n* = 11) and results may be biased due to an unknown number of participants also being part of the SMITH consortium. Formal validation of the TOP Framework’s performance against gold-standard phenotype definitions across diverse clinical datasets is essential. Although the correct functioning of the TOP Framework including the developed models is constantly evaluated on the basis of various fictitious data sets, we also plan to compare its accuracy, precision, and recall with established methods and benchmark datasets.

## Conclusions

The TOP Framework contributes to the field of phenotyping with a focus on user experience. The core concepts of the framework have been defined and its functionalities illustrated using the MELD score as an example. The MELD score demonstrates the TOP Framework’s capability to handle various data types and logical and computational expressions, making it suitable for phenotyping tasks. By providing a flexible platform for defining and querying patient cohorts in an intuitive manner, it has the potential to accelerate medical research and improve healthcare outcomes. The TOP Framework is utilised within the MII and has been evaluated with analyses from the large epidemiological study LIFE.

## Data Availability

The datasets supporting the conclusions of this article are publicly available under an open license [58]. A public demo instance of the TOP Framework is available at https://top.imise.uni-leipzig.de.

## Abbreviations

AKI: Acute Kidney Injury
BMI: Body mass index
CDM: Common data model
CI: Confidence interval
CQL: Clinical Quality Language
HER: Electronic health record
FDPG: Forschungsdatenportal für Gesundheit
FHIR: Fast healthcare interoperability resources
HL7: Health Level 7
INR: International normalised ratio
MELD: Model for end-stage liver disease
MII: Medical Informatics Initiative
NLP: Natural language processing
OLS: Ontology Lookup Service
PheMA: Phenotype Execution and Modelling Architecture
REDCap: Research Electronic Data Capture
TOP: Terminology- and Ontology-based Phenotyping
T2DM: Type 2 Diabetes Mellitus
UEQ: User Experience Questionnaire

## Footnotes

1 In this paper, we use the original version of the MELD score [18] as an example because it has been referred to in several studies and has already been used and published in different contexts. For the new MELD 3.0 [33], for example, no data are yet available on its correlation with the Child-Pugh score. However, it is absolutely possible to implement the new MELD version using our framework.

2 https://onto-med.github.io/top-deployment, accessed 09 Dec 2024

3 https://onto-med.github.io/top-deployment/administration/data-adapter-configuration, accessed 09 Dec 2024

## Declarations

### Project name

TOP Framework

### Project home page

https://onto-med.github.io/top-deployment/.

### Operating system(s)

Platform independent.

### Programming language

Java, Typescript.

### Other requirements

Java Runtime Environment version 17 or higher, Node.js version 18 or higher, software can be deployed with Docker.

### Licence

MIT.

### Any restrictions to use by non-academics

No restrictions.
